# Attitudes and preferences of traditional farmers toward reproductive biotechnology application for improved indigenous pig production in Zambia

**DOI:** 10.14202/vetworld.2022.403-413

**Published:** 2022-02-22

**Authors:** Rubaijaniza Abigaba, Pharaoh C. Sianangama, Progress H. Nyanga, Wilson N. M. M. Mwenya, Edwell S. Mwaanga

**Affiliations:** 1 Department of Animal Science, School of Agricultural Sciences, The University of Zambia, Great East Road Campus, Lusaka City, Zambia; 2 Department of Geography and Environmental Studies, School of Natural Sciences, The University of Zambia, Great East Road Campus, Lusaka City, Zambia; 3 Department of Biomedical Sciences, School of Veterinary Medicine, Great East Road Campus, Lusaka City, Zambia

**Keywords:** attitudes, indigenous pig production, reproductive biotechnology, survey, traditional farmers

## Abstract

**Background and Aim:**

Pig production remains crucial to the livelihood of farmers in Zambia. However, low production continues to undermine efforts to reduce animal protein deficit; hence, the need emerges to improve production through biotechnology. To contribute to the prediction of their acceptance, this study assessed the attitudes and preferences of traditional farmers toward reproductive biotechnologies by exploring the socio-demographic characteristics of farmers.

**Materials and Methods:**

The study conducted a cross-sectional descriptive survey that employed a mixed-methods design with a qualitative-quantitative methodological triangulation. Quantitative data were obtained from 622 respondents using a questionnaire, whereas seven focus group discussions (FGDs) were conducted to obtain qualitative data. Descriptive statistics and thematic analysis were used to analyze quantitative and qualitative data, respectively.

**Results:**

The majority (65.1%) of the respondents were low-income earners who mainly (64.8%) attained primary education. In addition, pig farming was dominated by middle-aged (43.7%) and elderly (40.7%) individuals. Moreover, most of the respondents owned (51.3% and 78.0%) more than 2 acres and six pigs, respectively. Furthermore, the respondents expressed a positive attitude (3.84±0.42) toward reproductive biotechnology application. However, despite supportive opinions (4.17±0.54) and favorable behavioral intentions (4.09±0.51), their feelings were generally neutral (3.10±0.89). In addition, the study observed various contrasting attitudes across socio-demographic factors. The respondents mainly preferred artificial insemination (AI; 66.2%). The results of the FGDs supported the survey findings. Nevertheless, the lack of information, knowledge and practical exposure, absence of peer influence, perceived beliefs and risks, poverty situations, and gender issues were pinpointed as the identified barriers to the biotechnology acceptance of the participants.

**Conclusion:**

The respondents generally supported reproductive biotechnology application and its contribution to improved production. However, further promotion of the favorable attitudes of the farmers will be required. In this case, interventions sensitive to their socio-demographic characteristics, perceived barriers, and identified contributing factors to favorable attitudes will be crucial. In addition, despite the overwhelming preference for AI, efforts to promote AI-supporting reproductive technologies are required because they contribute to AI success rate.

## Introduction

Food and nutritional insecurity remain a reality in sub-Saharan countries, including Zambia. For example, more than 40% of the rural population in Zambia lacks adequate food [1,2]. Moreover, the demand for food is predicted to triple by 2050; hence, an urgent need arises for more efficient food production systems with a particular emphasis on animal protein sources. The reason behind this notion is that the projected demand will largely depend on the increase in meat consumption with the increase in the human population [1,3]. Accordingly, policies that aim to diversify agriculture to include livestock production; promote improved production and productivity through biotechnology and conserve indigenous genetic resources have been ratified [1-4]. Among livestock species, pigs have been suggested for rearing as a strategy for reducing the animal protein deficit in the tropics [5-7]. However, to date, the majority of farmers (over 80%) continue to practice subsistence (traditional) farming, which is characterized by low production and fails to meet the available pork demand in Zambia [4,6,8]. For example, the country’s pork production in 2020 was 65,224 metric tons, which translates into a pork consumption per capita of 3.5 kg given the population of 18.4 million people [9,10]. Accordingly, this rate is generally low compared with the average global pork consumption per capita of 10.7 kg in the same year 2020 [11].

Therefore, the need and urgency for increased pig production that target indigenous pigs will be crucial for livelihood resilience among traditional farmers. Two strains of indigenous pigs, namely, Lusitu and Nsenga, are more adapted to local conditions and are reared by the majority of traditional farmers (65%) in Zambia [4,12]. To rapidly increase production, one of the means that traditional farmers must rely on is reproductive biotechnology utilization [13]. Biotechnology application improves reproductive efficiency, which is the backbone of enhanced animal production and productivity [14]. However, despite the widespread use and proven benefits of reproductive biotechnologies, such as artificial insemination (AI) and AI-supporting reproductive biotechnologies in many countries, their utilization in Zambia remains minimal or null among traditional farmers [14,15].

Furthermore, although the government intends to modernize farming for increased production, various issues, such as the lack of clear pig-breeding policies and the lack of knowledge about the biology of indigenous pigs, must be addressed first. In addition, introducing a form of biotechnology to the community is one issue; however, accepting or adopting it is another issue for farmers. Unfortunately, to date, a dearth of information exists about the crucial drivers of biotechnology acceptance and adoption rate, specifically, the attitudes of traditional farmers and the factors that influence such attitudes, such as socio-demographic and technology characteristics [16,17].

This study was carried out to generate attitude-related information from traditional pig farmers for policy and further research guidance. Accordingly, this study aims to (1) describe the selected socio-demographic characteristics of traditional pig farmers in Zambia, (2) assess the positive and negative attitudes of traditional pig farmers toward reproductive biotechnology application for improved indigenous pig production, and (3) establish the preference of traditional pig farmers for reproductive biotechnologies.

## Materials and Methods

### Ethical clearance and Informed consents

The study was carried out with approval (Approval number 1595-2021) by The University of Zambia Biomedical Research Ethics Committee (UNZABREC). All participants were informed about the study objectives and signed consent was obtained from them.

### Study period and area

**Figure-1:**
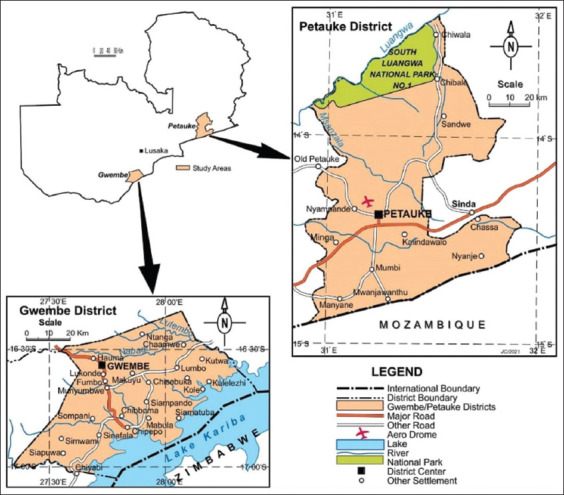
Location of the two study districts and agricultural camps from where respondents were selected. [Source: Department of Geography and Environmental Studies, University of Zambia].

The study was conducted in Gwembe and Petauke districts of Southern and Eastern provinces, respectively (Figure-1), from March 2021 to September 2021. The study areas have the highest pig population in Zambia [4,18]. Furthermore, Gwembe and Petauke districts are the areas of origin for Lusitu and Nsenga pigs, respectively. In Zambia, about 65% of the total national flock is comprised of indigenous pigs, namely, Lusitu and Nsenga. The other proportion is comprised of cross-breeds and exotic breeds, including Large white, Landrace, and Duroc [4,12]. Gwembe District lies within the agro-ecological region I while Petauke lies within agro-ecological region II. Their rain patterns and temperatures are 400-750 mm, 30-36°C and 750-1000 mm, 30-32°C for Gwembe and Petauke, respectively [4].

### Study design

The study conducted a cross-sectional descriptive survey that employed a mixed-method design to determine the socio-demographic information, attitudes, and understanding of farmers about reproductive biotechnology in relation to its application in pig production. The design employed a pragmatic approach for inter-subjectivity through the complementarity of the findings. Accordingly, quantitative and qualitative data collection methods were used with a major focus on the tripartite model of attitudes [19,21]. In addition, the qualitative-quantitative methodological triangulation was adopted to permit the breadth and depth of understanding about the subject matter and enable data collection within a short period [22,23].

### Sampling strategy

Traditional pig farmers were selected for the study using (1) the multistage purposeful random sampling strategy for the quantitative phase and (2) purposive sampling, which is based on the criterion-i sampling strategy, to obtain participants for the qualitative phase from the interviewed respondents during the quantitative phase. To ensure the validity of the results and minimize bias, the criteria used to recruit the participants included production system, gender, knowledge wealth on the subject matter, and pig breeds reared [22].

### Data collection and tools

Data on the socio-demographics and attitudes of the respondents were collected using the questionnaire survey and focus group discussions (FGDs). The design of the instrument for the questionnaire survey was based on previous studies related to biotechnology applications [24,25]. Concepts and items were modified with additions and omissions to suit the research topic. The tool design considered pragmatic opinions, feelings, and behavioral intentions; this was in consideration of the attitude complexity and multidimensional nature of the three components of attitudes, namely, cognition, affective, and behavior [21,25]. Accordingly, 25 items were rated using a five-point Likert-type scale, which captures various attitudinal aspects. In addition, multiple choice, dichotomous, and open-ended questions were included to collect socio-demographic data. Following a pre-test, Cronbach’s alpha indicated reliable internal consistency (a=0.767) for 18 items [26].

Likert-type items constituted the majority of the questions in the questionnaire. Therefore, the sample size for the questionnaire survey was estimated using a formula applicable at different population proportion levels and confidence levels [27]. The formula used was;


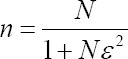


Where; n= minimum returned sample size; N=population size; ɛ=Adjusted margin of error which is
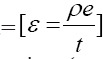
; *e*=degree of accuracy expressed as a proportion (margin of error at 0.03 for continuous data); *ρ*= number of standard deviations that would include all possible values in a range for a 5-point scale which is equal to 4; *t*=t-value for selected alpha level or confidence level=1.96 at 95% confidence interval. Other assumptions were; set alpha level a priori at 0.05, continuous data served a primary role in analysis, and population proportion estimate=0.5 was used since little was known about the study population [27,28].

Although the calculated sample size was 266, an adjustment by 134% increase to 622 was made to compensate for the design effect due to mixed methods at the multistage sampling. Out of 622 respondents, 269 and 353 were selected from Gwembe and Petauke district, respectively. A total of nine agricultural camps with six villages per camp on average were considered. From each village, 5 to 20 respondents were randomly selected.

FGDs or interviews (qualitative phase) were conducted using suitably constructed open-ended questions related to the subject matter. Based on the sampling strategy, the participants were invited using the phone numbers obtained during the questionnaire survey. A total of seven FGDs (three female groups and four male groups) were conducted, and each group was composed of 6-9 participants. The sample size and procedure used were mainly based on previous research [29,30]. Furthermore, all interviews lasted for 350 min, and each interview lasted for 40-60 min with an average of 50 min/interview. The interviews were audiotaped with occasional notes taken for non-verbal and verbal responses.

### Statistical analysis

Qualitative data from FGDs were transcribed, summarized, and categorized according to the main themes identified (thematic analysis), whereas the results were presented in the form of texts, illustrative quotes, and tables. Data from the questionnaire survey were analyzed by Statistical Package for the Social Sciences (SPSS) IBM^®^ (SPSS IBM 26 version, USA) using the basic descriptive statistics (measures of central tendency and variability) and principal component analysis (PCA). For attitude assessment, the reliability of the 18 items, which aimed to measure the overall or general attitude traits, indicated acceptable internal consistency (a=0.78). In addition, factor analysis (PCA with the oblimin rotation method) and reliability analysis (Cronbach’s alpha test) for the obtained variables were conducted to identify dimensions and determine the internal consistency of these variables (scales), respectively. The tests for sampling adequacy and the suitability of data for structure detection using the Kaiser–Meyer–Olkin (0.881) and Bartlett’s test of sphericity (χ^2^ [153]=4236.811, p<0.03), respectively, were suggestive of good data for PCA. Accordingly, conducting the PCA with the data provided a solution with three variables from the 18 items, which explained 58.40% of the variance.

Furthermore, the items with high loadings on the predetermined factors were selected and subjected to reliability analysis, which resulted in acceptable a values (Table-1). These items were used to further analyze attitudes with means and standard deviations obtained. The mean scores for attitude were computed by obtaining the total scores measured from the verbal responses of each respondent, which were rated using a five-point Likert-type scale (1=strongly disagree and 5=strongly agree) and divided by the number of items. The measuring scale for the mean scores was obtained by subtracting the smallest score (1) from the highest score (5) divided by 5 to obtain 0.8. This value was then added sequentially from the lowest score to the highest limit. In addition, Spearman’s rank correlation and Eta coefficient test were conducted to explore the association between the socio-demographics and attitudes of the respondents. Moreover, the study used frequencies to analyze the data on socio-demographic characteristics and biotechnology preferences. 

**Table 1:** Attitude variable description with scale name, item number, and scale reliability.

Attitude component or variable	Description of factors	Number of items	α
Cognitive	Only beliefs (pragmatic opinions) about biotech were considered.	6	0.78
Affective	Feelings in regard to emotions, worries, and unavoidability of biotechnology were key during item construction.	5	0.81
Behavioral	Since farmers had never used biotechnology, only behavioral intentions or willingness to use biotech were considered.	7	0.77

α=Cronbach’s alpha

## Results

### Demographic characteristics of the respondents

The majority of the respondents were female (66.9%). The majority (64.5%) completed the primary level of education. Among them, 65.1% reported a meager monthly income of <ZMW500 from all sources of household income. The middle-aged group was slightly more active in pig rearing (43.1%) than the other age groups. Many respondents (51.3%) had sufficient land (more than 2 acres) to undertake farming activities. The majority (65.9%) had an experience of <6 years in pig farming (rearing experience), whereas the majority (78.0%) of whom owned <6 pigs. Table-2 presents the socio-demographic characteristics.

**Table 2:** Socio-demographic characteristics of respondents.

Characteristic	Number interviewed	Percentage (95% CI)
Age of respondents (n=622)		
Below 30 years	101	16.2 (13.4-19.4)
30-45 years	268	43.1 (39.2-47.1)
Above 45 years	253	40.7 (36.8-44.7)
Level of education (n=622)		
No formal education	62	10.0 (7.7-12.6)
Primary	403	64.8 (60.9-68.5)
Secondary	154	24.7 (21.4-28.3)
Tertiary	3	0.5 (0.1-1.4)
Land or farm size (n=622)		
Below 1 acre	87	14.0 (11.4-17.0)
1-2 acres	216	34.7 (31.0-38.6)
Above 2 acres	319	51.3 (47.3-55.3)
Monthly income (n=622)		
Below ZMW 500	405	65.1 (61.2-68.9)
ZMW500-ZMW2000	158	25.4 (22.0-29.0)
Above ZMW2000	59	9.5 (7.3-12.1)
Flock size (n=622)		
Below 6 pigs	485	78.0 (74.5-81.2)
6-15 pigs	133	21.4 (18.2-24.8)
Above 15 pigs	4	0.6 (0.2-1.6)
Rearing experience (n=622)		
Below 6 years	410	65.9 (62.0-69.6)
6-10 years	92	14.8 (12.1-17.8)
Above 10 years	120	19.3 (16.3-22.6)

CI=Confidence interval, US$1=ZMW16.50 (29 September 2021)

### Attitudes toward reproductive biotechnology application

The verbal responses of the respondents to all items on attitudes were computed to obtain the nature or the mean score of their general attitude. Accordingly, the mean score (3.84±0.42) revealed generally positive attitudes toward reproductive biotechnology utilization if rated on a scale of 1 to 5 (1.00-1.80=strongly negative, 1.81-2.60=negative, 2.61-3.40=neutral, 3.41-4.20=positive, and 4.21-5.00=strongly positive). Furthermore, the computed mean scores for the three other scales, namely, opinions, feelings, and behavioral intentions, pointed to positive (4.17±0.54), neutral (3.10±0.89), and positive (4.09±0.51) evaluations, respectively.

### Attitudes across socio-demographic characteristics

The verbal responses for the overall attitudinal evaluation were computed across each socio-demographic characteristic to explore the nature of the attitudes of all sub-groups (categories). The mean scores for attitude obtained using a scale of 1-5 (the lowest and highest values indicated negative and positive attitudes, respectively), indicated positive attitudes for male (3.91±0.43) and female (3.81±0.40) farmers. The study observed no association (eta=0.113) between a change in attitude and gender predisposition. Table-3 presents the mean attitude evaluations across other characteristics. In this case, attitudes across education, age, income status, land size, rearing experience, and flock size were positive for all categories. Table-4 depicts those changes in attitudes were positively associated with level of education (p<0.01), income status (p<0.03), number of pigs owned (p<0.01), years of rearing experience (p<0.01), and size of land owned (p<0.01). Coincidentally, although statistically non-significant (p>0.03), the study observed a tendency toward a negative association between age and change in attitude.

**Table 3:** Respondents’ mean attitude scores across sociodemographic characteristics.

Characteristic	Frequency	Mean±SD
Education level (n=622)		
No education	62	3.67±0.45
Primary	403	3.81±0.40
Secondary	154	3.96±0.39
Tertiary	3	4.51±0.51
Monthly income (n=622)		
Below K500	405	3.83±0.37
K500-K2000	158	3.85±0.52
Above K2000	158	3.90±0.40
Years rearing pigs (n=622)		
Below 6 years	410	3.78±0.43
6-10 years	92	3.93±0.42
Above 10 years	120	3.97±0.32
Age (n=622)		
Below 30 years	101	3.82±0.47
30-45 years	268	3.84±0.41
Above 45 years	253	3.84±0.40
Land size (n=622)		
Below 1 acre	87	3.77±0.42
1-2 acres	216	3.78±0.43
Above 2 acres	319	3.90±0.40
Flock size (n=622)		
Below 6 pigs	485	3.81±0.43
6-15 pigs	133	3.94±0.35
Above 15 pigs	4	4.05±0.41

Mean scale: 1.00-1.80 (Strong negative), 1.81-2.60 (Negative), 26.1-3.40 (Neutral), 3.41-4.20 (Positive), 4.21-5.00 (Strong positive), SD=Standard deviation

**Table 4:** Association between respondents’ mean attitude scores with socio-demographic characteristics.

Association (Correlation)						

Characteristic	Education Level	Age	Income status	Rearing experience	Land size	Flock size
Attitudes	r=0.225** (Positive)	−0.190 (Negative)	0.089* (Positive)	0.183** (Positive)	0.147** (Positive)	0.133** (Positive)

Spearman rank correlation coefficient (r_s_), Superscript

** or

* indicate statistical significance at *p <*0.01 or p*<*0.03, respectively.

### Preference for reproductive biotechnologies

The respondents were asked to recommend innovations that they felt should be applied. AI was the most favored biotechnology (66.2%), whereas *in vitro* fertilization (IVF) and embryo transfer were the least preferred (Table-5). When asked about their opinion on government plans to modernize livestock production, 85.3%, 10.0%, and 4.7% were in support of, neutral about, and against government plans, respectively.

**Table 5:** Respondents’ preference for biotechnology.

Preferred biotechnology	Frequency (%)
Artificial insemination	412 (66.2)
OI and OS	36 (5.8)
Semen preservation	15 (2.4)
Pregnancy diagnosis	37 (5.9)
Semen evaluation	17 (2.7)
IVF and embryo transfer	14 (2.3)
No answer	91 (14.6)
Total	622 (100.0)

OI and OS: Estrus Induction and Synchronization, IVF=*In vitro* Fertilization

### FGDs

FGDs were conducted to shed light on the results of the questionnaire survey and obtain additional views and opinions from the respondents about reproductive biotechnology application. The major themes that emerged from these interviews included (i) general view about these innovations, (ii) potential barriers to favorable attitudes toward and acceptance of reproductive biotechnology, and (iii) possible factors that facilitate favorable attitudes toward reproductive biotechnology.

### Perspectives and general views about reproductive biotechnology application

The respondents reported that they would accept these innovations given their potential benefits. They believed that utilizing these innovations would be a panacea to their nutritional and monetary household needs. Moreover, they intended to increase pig production because pigs are used to generate money for school fees, fertilizers for crops, food, medical bills, and a source of meat for home consumption. Thus, the respondents held various supportive views regarding the use of reproductive biotechnologies. We present several responses that are worth quoting in terms of specific biotechnology.

*Ah… yes, the methods especially AI can help us even to cross local breeds with exotic ones so as to improve the size of our local pigs… but this should be noted, local pigs are disease resistant!*
*… imagine all the 10 pigs can be put on heat at the same time and you expect about 50 piglets, this would be good for me as a farmer to get out of poverty …*.
*I have two methods that have impressed me and these are; heat detection and embryo transfer. This is because I would not wait for so long to breed and get piglets, for us with our traditional ways we take longer. Even for embryo transfer, it can be done within a short time and it will lead to realizing pigs or benefits faster*.

Furthermore, the study identified several reproductive biotechnologies that were favored by the participants, including their perceived advantages or benefits (Table-6).

**Table 6:** Participants’ preference for biotechnology and perceived advantages.

Preferred biotechnology	Perceived advantage
Artificial insemination (AI)	Improve indigenous pig size through cross-breeding. Facilitate breeding of sows. Reduced reproductive losses by reducing farrowing interval. More money because many piglets are produced. AI is cheaper than keeping boars. AI allows breeding anytime and when needed.
Estrus induction and synchronization	Shortens farrowing interval. Reduced poverty when more pigs are produced.
Embryo transfer	Reduced farrowing interval. More money since more pigs are produced in a short time.
Heat detection	Reduced farrowing to the breeding interval. More pigs produced hence more money per year.
Semen preservation	AI allows breeding anytime and when needed.

The participants exhibited a tendency to compare the traditional and modern methods (reproductive biotechnologies) of pig production. Notably, their feelings and perception about traditional methods were unfavorable. As such, they favored the reproductive biotechnologies to increase production of their indigenous pigs, which they considered low and undesirable.

*We have had our local pigs for a long time but without increasing the number significantly and we would like to embrace these methods to increase our pigs and also hope that the challenge of African swine fever is reduced when we use AI. Yes, because with AI, semen evaluation is done so that only semen without disease is used. Ah… also, AI will allow us without boars, to breed our female pigs*.

Furthermore, they pointed to the traditional method as the crucial factor facilitating disease spread.

Although the participants were not utilizing modern biotechnologies, they expressed admiration for people and countries that are currently utilizing them and wished to do the same. They expressed concern over the low production of indigenous pigs despite their value. Many respondents were aware that other countries had increased pig production with the application of reproductive biotechnologies and expected similar results once the same are benchmarked. Furthermore, they wished to share and learn from such countries to change their mindset.

*Of course, we support their idea of using these methods, but we also want to use them*.
*If other countries are using them, we can also do the same so as to expand our pig production business. When we start, others will follow us and then increase our pig production, get food, and change our lives, our community, and the country*.

### Barriers to acceptance of reproductive biotechnology application

During the FGDs, the participants reported that these reproductive biotechnologies are good; however, many issues may lead farmers to refuse these methods. In particular, two participants referred to the absence of peer influence as a potential barrier:

*Naturally, farmers want to just see others using certain methods and then they also follow*.
*Yes, there are things us as human beings that we don’t appreciate at the beginning until we see the benefits our friends are ripping out of doing them and then we also accept to do them*.

Moreover, the study identified other barriers and reasons, which may likely deter favorable attitudes toward these reproductive biotechnologies (Table-7).

**Table 7:** Potential barriers to acceptance of reproductive biotechnology application.

Barrier	Reason
Inability to appreciate the benefits of biotechnology.	Lack of information and knowledge
Failure to appreciate their practicality.	Lack of practical exposure
Failure to appreciate physical benefits.	
Lack of advice and persuasion from fellow farmers.	Absence of peer influence
Failure to appreciate demonstrable benefits.	
Fear for or the possibility of a loss of their pig breeds.	Beliefs and perceived risks
Ungodly nature of biotechnology application.	
Uncertainty over their practicality in indigenous pigs.	
Perception of unaffordability of biotechnology services.	
Inability to pay for biotechnology, feeds, and veterinary services.	Poverty situations
Resistance from men undermines acceptance by females.	Gender issues

### Factors contributing to favorable attitudes toward reproductive biotechnologies

This section discusses the perspectives and views of the participants about the possible contributing factors to favorable attitudes. The participants reported that routine sensitization and education about these innovations and emphasis on the benefits accrued from their application were deemed to be extremely important. Hence, stakeholders should employ various possible means, such as regular meetings and home or farm visits, to avail information and impart useful knowledge about the same to farmers.

*Many times if a person doesn’t know anything, he or she can’t accept.… what is important is that one needs to learn and have more knowledge so that we can be able to accept it wholeheartedly.… also we people have a mentality of wanting to do things habitually so that it is easier to accept and many people will join*.

Apart from this view, the respondents further elaborated that practical exposure by implementing the innovation in the community in the form of demonstrations may help or entice farmers to favor these methods after witnessing their viability and tangible benefits. Moreover, the farmers pointed out that they would like the government to promote a farmer-to-farmer peer approach to attitudinal change through the establishment of model farmers in the community to appreciate these innovations and become inspired practically.

The participants reported that one of the best approaches to influencing favorable attitudes is the implementation of the government policy on biotechnology. The farmers believed that once this government policy is implemented, several farmers will promptly favor it. Naturally, the rest will also develop positive attitudes when they witness the progress of others. In addition, they mainly believed that poverty was another important factor that may influence their acceptance rate. They explained that poverty situations can always force one to accept change to escape poverty. Thus, the government should target the poor.

*Ah yes, government should start mainly to target us the poor, us who are poor are willing to use these biotechnologies so that we can improve our lives*.

## Discussion

Although the need for reproductive biotechnologies and government plans to promote their utilization are existing, policy framers and implementers must be able to predict the extent of the acceptance or adoption of farmers [31]. Toward this end, one needs to obtain information about the attitudes of farmers because their attitudes influence the widespread acceptance of new innovations [32]. Our quantitative assessments with corroboration from qualitative analysis revealed general positive attitudes toward biotechnology use. The general attitudes were positive, and the component evaluations based on the tripartite approach with a focus on a 3D model [21] revealed positive opinions and behavioral intentions. However, the feelings were generally neutral. This finding indicated that a conflict occurred between the opinions and feelings of the respondents; however, their opinions were dominantly supportive over feelings, which culminated into favorable behavioral intentions toward biotechnology use. A possibility is that the perceived benefits from reproductive biotechnology use, which were identified during the FGDs, influenced the increased supportive opinions of the respondents; previous scholars reported that perception influences attitudes toward biotechnology [16]. However, the neutral feeling may be attributed to perceived risks and negativity related to biotechnology [32]. Therefore, once attention is given to the nature of the explored triodes as well as the views and perspectives of the respondents, then policy actors will likely benefit from the findings of this study.

### Demographics and attitudes toward biotechnology application

To facilitate innovation acceptability and ensure a result-oriented intervention, appropriate consideration of demographic factors and attitudes is necessary [17,31,32]. Toward this end, the study explored socio-demographic and attitude evaluations across these factors. With regard to gender, the dominance of females in keeping pigs concurred with the findings of previous studies in South Africa (64.9%) and Botswana (62%) [33,34]. The dominance of the females could be attributed to the low inputs required, the backyard approach to rearing, and the small size of pigs compared with cattle, rendering pig rearing manageable for females. In addition, men may despise pig rearing and regard it as a minor activity given the patriarchy societal setting. Thus, men mainly leave this venture to women. Regardless, the observed female dominance was supportive of the present efforts to engage women in pig production for income generation and support their role in families and the community [7]. In contrast, although the males were less dominant, they exhibited more favorable attitudes toward biotechnology than the females. This finding is in support of a previous research [32]. Furthermore, despite the lack of statistically significant association between gender and attitudes in the current study, previous scholars linked gender to risk and benefit perception [32].

The generally low education status of the respondents was similar to that of a previous study in India, which reported that the majority of the respondents (90%) that rear pigs achieved basic primary education [35]. In general, farmers with low levels of education are not permanently employed in the formal sector. Thus, such people are mainly dependent on animal production for their livelihood [34]. In this case, the findings from the FGDs regarding the role of pigs in their lives support the narrative. In this view, exerting effort to increase production in order to promote the improved livelihoods of farmers was deemed crucial. Cognizant of the role of biotechnology in improving production, the attitudes of the respondents across levels of education was assessed to explore the potential for biotechnology acceptance. This study demonstrated that the respondents exhibited favorable attitudes toward biotechnology and supported the need to modernize livestock production [5,8]. In addition, the observed positive change in attitude with the high levels of education was in line with the findings of previous research, which indicated that the education factor influences attitudes toward biotechnology [32]. Even if the unschooled exhibited positive attitudes, their mean score compared with educated ones or those with higher levels of education, supported the existing notion that farmers with high levels of education are more active in knowledge seeking due to their ability to appreciate the benefits of reproductive biotechnologies [31,32]. Nevertheless, the participants also underscored the importance of sensitization and education in facilitating favorable attitudes.

The observed dominance of the middle-aged category (30-45 years) concurred with previous findings in India, where the majority of farmers (60%) aged 30-40 years were more involved in pig farming [35]. The dominance of the middle-aged category may be attributed to the existing notion that middle-aged people tend to have families and shoulder responsibilities. Thus, they rear pigs as a source of income and meat for home consumption [33,34]. Needless to say, the FGDs pointed out these roles as the main motivation for indigenous pig rearing. In addition, a previous study in Botswana attributed the perception held by the younger people, that is, pig rearing is for the older people, to their minimal participation. However, the lack of capital may also likely contribute to this notion [34].

Nevertheless, and in light of the need for farmers to adequately attend to their household needs, production must increase by means of biotechnology [13,14]. In general, farmers in the middle-aged category tend to help in technology transfer because they are flexible to new technologies [36]. Coincidentally, the current findings illustrated a slightly higher mean attitude score for this category compared with the elderly and the younger age group. This reality, higher attitude scores among the middle-aged category than the elderly and younger age groups, was reportedly responsible for the weak association between respondents’ age group and their attitude evaluation. Moreover, the previous studies in Tunisia and Malaysia reported the negative influence of age on the attitudes of farmers [37,38].

During the FGDs, the respondents pointed out their poverty situations. This notion supported the quantitative finding that pointed to the dominance of low-income earners in pig rearing. Given an income index of 1.25 USD/capita income/day, these respondents were living below the threshold of poverty. The current findings concurred with those of the previous studies in India, where 85% of the farmers were poor [35,39]. Furthermore, the respondents reported pigs as their key source of income and meat for home consumption, which were similar to roles reported by a study conducted in Vietnam [39]. The observed positively changing attitudes with higher income categories supported the existing findings that reported the positive influence of income on the attitudes toward and adoption of biotechnology among farmers [32,37]. According to Moon and Balasubramanian [32], income is related to the financial ability to access good information. Notably, even the low-income earners displayed a favorable attitude, which may be linked to the perceived benefits of reproductive biotechnology and their desire to escape poverty. Nonetheless, the wish of the participants for the government to subsidize or provide free biotechnology services remains an issue that warrants further attention.

To promote the livelihood of traditional farmers, issues on land ownership and farm size require consideration. In line with this notion, the present study demonstrates that many respondents owned sufficient land which can facilitate pig production. This finding disagreed with those of the previous studies in India, where 85% of the pig farmers were landless [35]. Furthermore, although this study indicated that only 14.0% of the respondents owned <1 acre or no land, the previous findings confirmed that land ownership positively affected the probability of a farmer accepting or adopting an innovation [40]. Notably, pigs can be reared on a small piece of land because they are not grazers and are regarded as the most ideal to rear on given the space requirement [41]. Nevertheless, even if land size may not necessarily be of great concern to a pig farmer, especially in the rural setting, this study found that the more land a respondent owned, the more positive attitude evaluation is observed.

In the current study, the observed majority of respondents with <6 years of rearing experience disagreed with the study in Cameroon, where farmers had a longer rearing experience of 10 years [41]. A possible reason is that worsening economic situations and nutritional demands and changes in the mindset of the farmers in terms of gender roles may have promoted the recent widespread involvement of people in pig farming. Despite this scenario, the small proportion of respondents with more than 10 years of rearing experience did not match with the fact that the Lusitu and Nsenga breeds of pig have historically existed in these areas, including the value that farmers have long ascribed to these indigenous pigs. Nevertheless, the FGDs demonstrated the motivation of the participants for pig rearing, including the value attached to indigenous pigs. Although the respondents across all categories exhibited positive attitudes; their attitude evaluation was found to be more positive with the increase in rearing experience. Another possibility may be more favorable attitude evaluations due to many years of experience were related to the cumulative information about the benefits of biotechnology, which has been acquired over time. These results are consistent with the previous report [31], which indicated that traditional farmers in the rural setting are commonly associated with longer awareness phases.

The existing reports indicate that the majority of pig farmers rely on informal employment sectors for their livelihood [34,41]. Unfortunately, although the participants (in FGDs) underscored the importance of pigs, the quantitative exploration demonstrated that the majority owned <6 pigs. Nevertheless, the findings were similar to those of a previous study in Vietnam, which reported that 87% of pig farmers owned <5 pigs [39]. Moreover, ownership of a small flock size has been linked to various factors, such as lack of proper veterinary services, lack of good breeding boars, inbreeding, lack of technical knowledge on production, financial inability to expand business, diseases, and feed scarcity [39]. Similarly, these factors were also pointed out during our FGDs. Nonetheless, the observed favorable attitudes of the respondents with <6 pigs were suggestive of potential acceptance if the government was to implement the policy on biotechnology. Moreover, the observed attitude was even more favorable among respondents with more than six pigs.

### Reproductive biotechnology preference

Although many reproductive biotechnologies have been innovated, not all of them are widely or globally utilized. Moreover, the adoption rate is lower in African countries [13,15]. To facilitate their widespread adoption, various issues, such as socio-cultural characteristics, will require attention, because they influence the exposure to innovations and perceived benefits or risks. In turn, this information will influence the technological choices of farmers [16,31,32]. In this case, determining the position or preference of farmers in the face of a technological offer is crucial to predict better as well as influence biotechnology acceptance or adoption [42]. In contribution to this aspect, the current study found that the majority of respondents favored AI more than they did for other innovations. Nevertheless, AI was also previously reported as the most widely utilized innovation in animal production globally [13,15]. Thus, a possibility exists that the perceived benefits of AI and increased knowledge about the same compared with other innovations contributed to the magnitude of preference for AI in the current study.

Although quantitative analysis depicted AI as the most favored biotechnology, the FGDs revealed a considerable preference for estrus induction and synchronization, heat detection, and embryo transfer. The findings are in agreement with those of existing studies that ranked synchronization and embryo transfer as the second and third widely used methods, respectively, after AI [13,15]. Nevertheless, the less popularity of these reproductive biotechnologies was probably due to the lower awareness of the respondents about these reproductive biotechnologies compared with AI. This finding is in view of the fact that people form attitudes when they acquire relevant information or become aware of an innovation [16,25,31]. In addition, the study pinpointed other issues, such as uncertainty about their practicality and associated socio-cultural beliefs, as identified in the FGDs, because the previous studies associated them with the attitudes of farmers toward biotechnology application or adoption rate [13,15,16,42]. Regardless, AI-supporting reproductive biotechnologies require due attention from policymakers and implementers in light of their role in ensuring the success rate of AI. The respondents overwhelmingly supported government plans to utilize biotechnologies. This finding was suggestive of the potential for reproductive biotechnology acceptance once introduced. However, the extent may depend on the level of attention given to the identified perceived barriers and suggested approaches to facilitating positive attitudes among farmers.

## Conclusion

In light of the crucial roles of pigs in the livelihood of traditional farmers, biotechnology application has been deemed a means that must be used to improve production. Furthermore, this study found that mainly women farmers, low-income earners with low levels of education, owning small flock sizes of pigs, and with <6 years of rearing experience of pig rearing. In general, the respondents exhibited positive attitudes toward reproductive biotechnology application. Notably, however, the feelings of the respondents were generally neutral despite the favorable assessment of their opinions and behavioral intentions toward biotechnology use. In addition, although AI was the most favored method, the findings of the FGDs revealed the potential for acceptance and widespread popularity of several AI-supporting reproductive biotechnologies given due attention to the views and perspectives of the participants regarding the perceived barriers and approaches to more favorable attitudes.

This study has its limitations. First, attitude is a complex construct comprised of many factors within components. Thus, its precise measurement is problematic. Second, the construction of the questionnaire mainly excluded items for measuring knowledge and concentrated on opinions for the cognitive component on the assumption that the respondents were not using reproductive biotechnologies. Third, the sample size and study area considered may influence generalizability and reproducibility of the findings in urban areas, for instance. Therefore, using a larger sample size drawn from various areas or districts, including urban areas, and employing a richer set of questions that are designed to capture various factors within each attitude component will be crucial for future studies that intend to replicate the current study. This recommendation is in view of the impact of the aforementioned aspects on total variance explained by attitude components, which, thereby, render the current findings more generalizable. Regarding the observed uncertainty over the practicality of the use of reproductive biotechnologies on indigenous pigs, comparative studies that focus on the reproductive biology of the Lusitu and Nsenga strains and their potential improvement through the utilization of reproductive biotechnologies are required.

## Authors’ Contributions

RA: Study conception, design, data collection and analysis, and drafted the manuscript. PCS, ESM, PHN, and WNMM: Study design, supervision of study implementation, and manuscript review. All authors read and approved the final manuscript.
